# *Voyager*: rapid taxonomic characterization of long-read mixed-species sequencing samples in sorted motif distance space

**DOI:** 10.1186/s13059-026-04235-3

**Published:** 2026-08-05

**Authors:** Sverre Branders, Manfred G. Grabherr, Rafi Ahmad

**Affiliations:** 1 Department of Biotechnology, University of Inland Norway, Holsetgata 22, Hamar, 2317 Norway; 2 Institute of Clinical Medicine, Faculty of Health Sciences, UiT - The Arctic University of Norway, Hansine Hansens veg 18, Tromsø, 9019 Norway

**Keywords:** Long-reads, Metagenomics, Microbiome, Deconvolution, Alignment-free methods

## Abstract

*Voyager* is a novel algorithm that complements real-time sequencing by rapidly mapping long sequencing reads with insertion and deletion errors to a known database of organisms. It utilizes *Sorted Motif Distance Space*, distances between exact matches of short motifs sorted by rank, to represent sequence similarity in a highly compressed, computationally efficient manner while enabling strain-level discrimination. *Voyager* incorporates a deconvolution algorithm to discern closely related genomes. It achieves an average species-level accuracy of 99% across 15 datasets, outperforming all evaluated methods. *Voyager* maintains CPU runtimes comparable to the current fastest methods while using less than half the memory.

**Supplementary Information:**

The online version contains supplementary material available at https://doi.org/10.1186/s13059-026-04235-3.

## Background

Long-read sequencing is a nucleic acid sequencing method that allows for the sequencing of much longer fragments (thousands of nucleotides or more) than traditional short-read sequencing methods. Currently, two leading long-read sequencing technologies are Pacific Biosciences (PacBio) and Oxford Nanopore Technology (ONT). The journey to the next generation of medical care in the face of infection started with ONT, providing sequencing data in near real-time parallelly with an ongoing sequencing run rather than after a completed sequencing run, as with previous generation methods. This capability, combined with its portability, low cost, and ease of library preparation, opened a path to the use of sequencing technology in routine diagnosis [1]. Long-read sequencing technology, especially PacBio sequencing, already plays an important role in the surveillance of foodborne pathogens [2, 3]. For time-sensitive applications, rapid analysis of long-read data may be vital. In a clinical context, quick analysis of samples obtained from patients’ bodily fluids, e.g., blood, sputum, urine, etc., to characterize pathogens will lead to faster and informed treatment, reduced patient time in the hospital, and improved patient outcomes [1]. Likewise, fast and user-friendly characterization of infections in the veterinary and livestock sector can relieve the need for preventative antibiotics and prevent the spread of infection to the rest of the herd [4]. To arrive at such new rapid clinical diagnostics, novel computational methods are needed to characterize known pathogens quickly and accurately and take advantage of these real-time capabilities.

Previously, the problem of identifying species in a mixed-species sequencing sample has been addressed by several methods, including mapping the reads to a set of known reference genomes in nucleotide space [5–9], in amino acid space [10–12], or by identifying marker genes [13, 14], by, e.g., analyzing the local nucleotide characteristics against a reference database [8, 9]. The fastest of these methods, such as kraken2, rely on matching up short consecutive strings of nucleotides (k-mers)^13^, allowing for fast look-up or for analyzing k-mer frequencies, compensating for isolated sequencing errors in between perfect matches. More recently, methods that rely on compressed representations of sequences have become more widely used for characterizing metagenomic sample composition, using tools such as sourmash and sylph [15, 16]. While metagenomic methods are especially well established for short read sequencing methods, such as Illumina, where errors manifest themselves predominantly as single nucleotide substitutions, there remains a need for algorithms robust to long reads with insertion/deletion error patterns commonly found in Oxford Nanopore Technologies (ONT) sequences data [17]. Initially, ONT sequencing had an error rate of over 10%, with most errors caused by insertions or deletions [18]. In recent years, the error rate has steadily decreased. However, most remaining errors are still caused by frequent short deletions or, less frequently, long insertions [19].

Here, we present *Voyager*, a new method that maps reads in *Sorted Motif Distance Space* (*SMDS*) and infers the taxonomic composition of a sample containing known organisms from these mappings across a complete sequencing run.

## Results

### Novelty

**Fig. 1:**
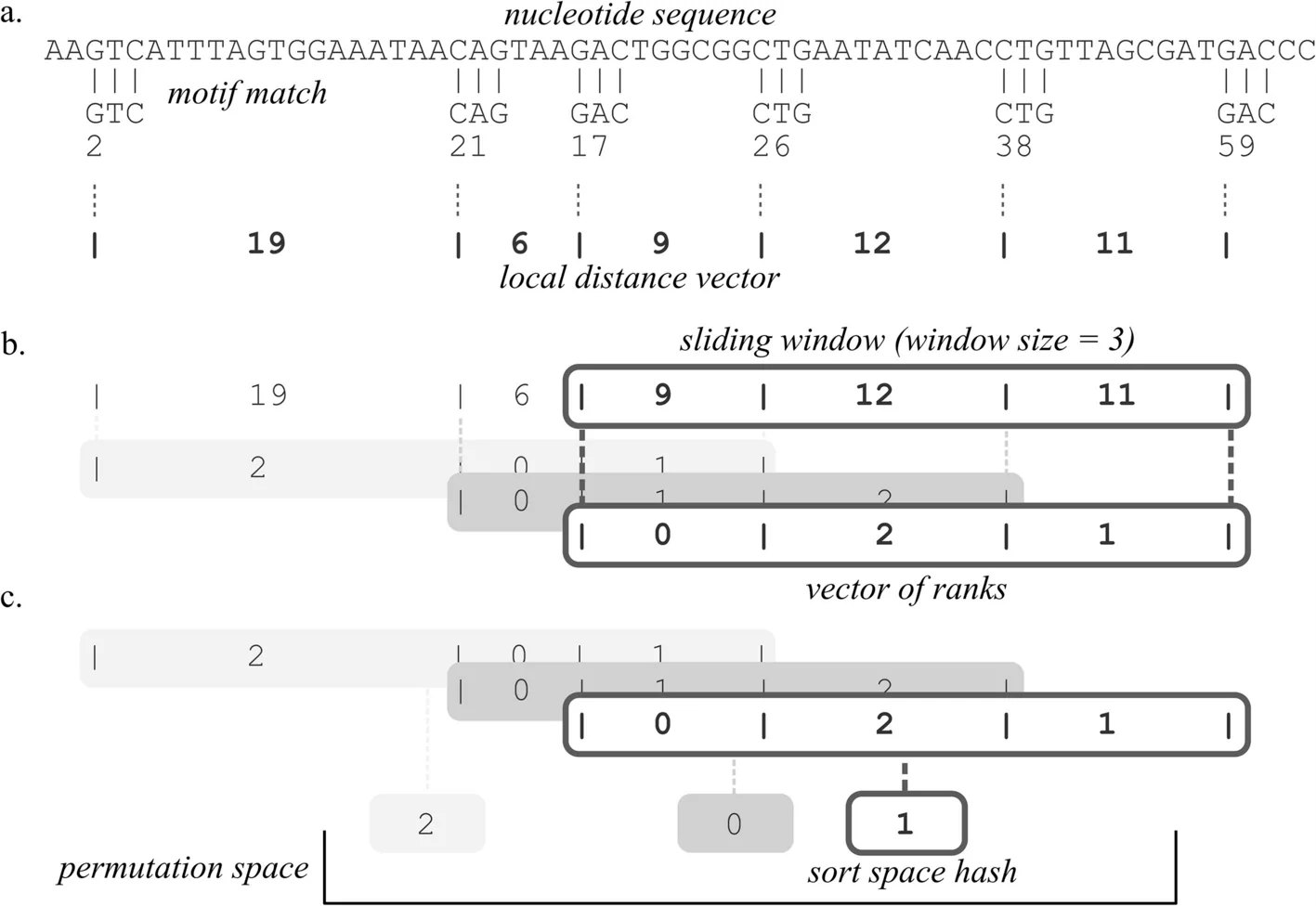
Schematic of the Voyager algorithm. **a** The local nucleotide distance between exact matches of a soft-mer motif (here, GWC) is measured to construct a *local distance vector*. **b** A sliding window approach with a given *window size* (here, 3) is applied to the *local distance vector*. Within each window, the local distances are ranked in ascending order to obtain *vectors of ranks*. **c** A unique *Sort Space Hash* is computed for each unique *vector of ranks*. These hashes are used to construct the *Permutation Space* of a genome and subsequently for rapid matching of query sequences using a hash map data structure

*Voyager* works by measuring the distances between exact matches of short motifs (Fig. 1a), implementing a concept that borrows from optical restriction map alignments [20]. Unlike k-mers, i.e., local short consecutive sequences, these distances span dozens, hundreds, or even thousands of nucleotides, depending on the choice of the motif. When combining consecutive distances *into local distance vectors* of a certain fixed *window size* (Fig. 1b), these vectors are highly specific to the sequences they originate from since the exact distances between motifs are unlikely to occur by chance and in the same order in a different genome.

To map error-containing sequencing reads, instead of using distance vectors directly, *Voyager* sorts these vectors into *vectors of ranks* (Fig. 1c), effectively making the ranks (the positions of the values in a sorted vector) the key for look-up in a hash map: the ranking is preserved in the presence of insertions and deletions, so long as the sorted order is not changed. Given a fixed window size *n*, the number of possible permutations is *n* factorial, e.g., for *n* = 14, there are 8.71 × 10^10^ permutations. When using a motif that occurs every 100 nucleotides on average, this *permutation space* is about three orders of magnitude larger than the number of *vectors of ranks* found in a mammalian genome (e.g., 9.97 × 10^7^ in the human genome) and still two orders of magnitude above large plant genomes. Moreover, the ranked vectors obtained from a variety of genomes appear to be evenly distributed, suggesting effective use of the *sorted motif distance space* for hashing (Additional file 1).

As a result, *Voyager*’s algorithm (a) is computationally efficient due to a sparse and long-range search space; (b) compresses the search index, thus memory usage, by omitting the sequences in between motifs and only using the distances; and (c) provides high specificity, to the degree that individual strains are discernable by applying a novel approach to correct for false-positives resulting from sequence complementarity. The observed mappings from a sample are an indirect function of the underlying composition of genomes in the sample and the sequence complementarity of those genomes- in SDMS—thus *Voyager* applies a deconvolution step, in which the signals from genomically similar sequences are corrected for. If, for example, two closely related organisms share sequences, then deconvolution will resolve which organism is more likely to have contributed to a read, without eliminating the possibility that both organisms are present. For making this algorithm robust, we apply the non-negative-least-squares (NNLS) algorithm [21], which ensures that read assignments to genomes always result in estimates equal to or greater than zero.

In the following, we describe the algorithms in detail, explain our choice of parameters, and then demonstrate how this conceptually simple but innovative algorithm compares against existing methods.

### *SMDS* provides an efficient and specific mapping algorithm

**Fig. 2:**
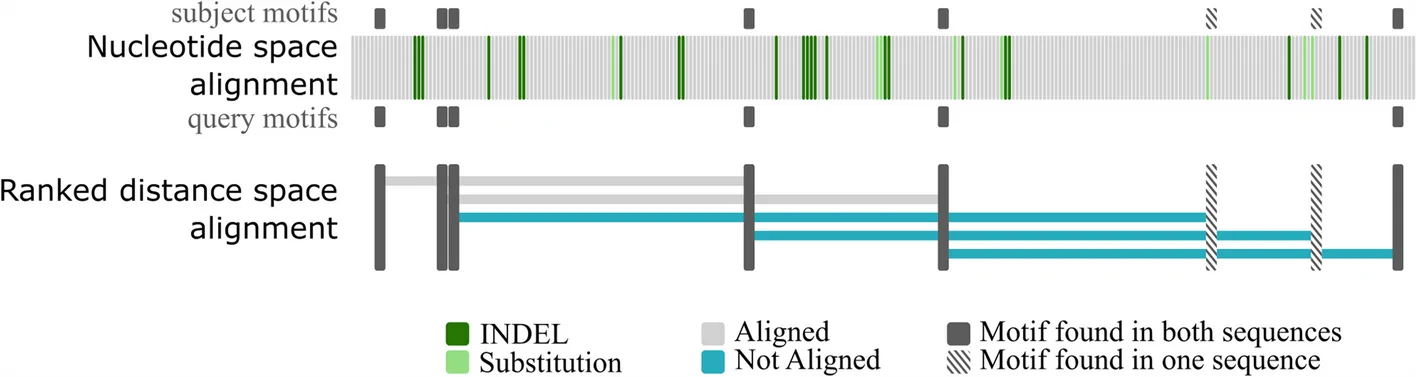
Illustration of the *Voyager Sorted Motif Distance Space* matching algorithm. The figure shows an alignment of a sequence (subject) to an error containing ONT read (query) of the corresponding gene in nucleotide space. Inserted or deleted nucleotides are indicated in dark green, and substituted nucleotides are indicated in light green. Gray blocks indicate where matches of motifs, used by the *Voyager* algorithm, are located on the subject and query sequences. Two motifs are present in the subject, but not in the query, as a result of substitution errors (hashed block). Below, the same aligned region is represented in *Sorted Motif Distance Space (SMDS)* (*window size* = 4). Because of the two missing motif matches in the query sequence, three of the five *Sort Space Hashes* no longer match in SMDS

The working mechanism of *Voyager*’s *SMDS* mapping algorithm is further illustrated in Fig. 2. The Figure shows a 274 bp section of an alignment of a query sequence containing six motif matches to an ONT sequencing read of the corresponding gene region. In nucleotide space, the alignment includes 23 INDEL mismatches and eight substitution mismatches. Due to substitution errors, the query sequence is missing two motif matches. Despite the high INDEL errors in the ONT read sequence (given a window size of four), two of *the ranked distance vectors* are perfectly conserved. Where errors affect motif matches, the sorted order changes in the windows containing these, and they thus no longer match. This mechanism gives *Voyager* its discriminative ability.

**Fig. 3:**
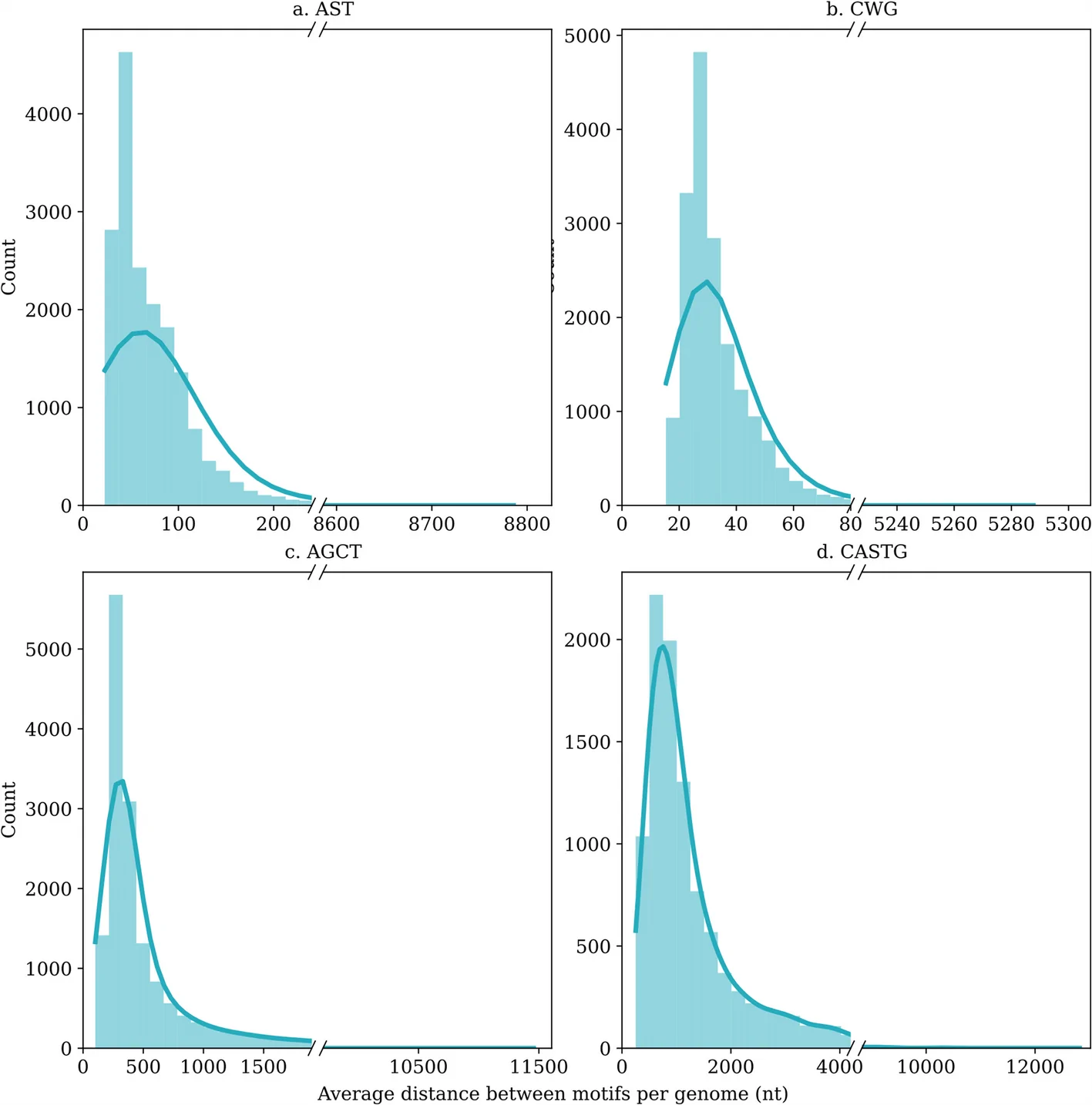
Histograms of the average distances between motif matches. The histograms show the average distance in nucleotides between exact motif matches for all genomes in the NCBI RefSeq prokaryotic representative genomes collection. The distances were obtained using different motifs: **a** AST, **b** CWG, **c** AGCT, and **d** CASTG

Moreover, the *ranked distance space* greatly compresses the search space. When constructing *local distance vectors* of the NCBI RefSeq [22] prokaryotic Representative Genomes using the soft-mer motif “AST” (where “S” is either C or G), on average, the distance between motifs is 106 nucleotides (σ 328) with 95% of the distances falling between 30 and 329 nt (Fig. 3a). The average distance between motifs “CWG” (“W” is A or T) (Fig. 3b) is 38 nt (σ 74), with 95% of distances between 19 and 86 nt. When using motif “AGCT” (Fig. 3c), the mean distance increases to 658 nt (σ 868) with 95% of distances above 183 nt and below 3374 nt. Motif “CASTG” (Fig. 3d) results in a mean distance of 1407 nt (σ 1259) with 95% in the range of 393 nt to 5088 nt. Since the ranks can be represented by 8-bit integers, Voyager allows for storing this genomic information by omitting the nucleotide sequence in between motifs efficiently, i.e. in a single byte.

### *Voyager* achieves strain level specificity

**Fig. 4:**
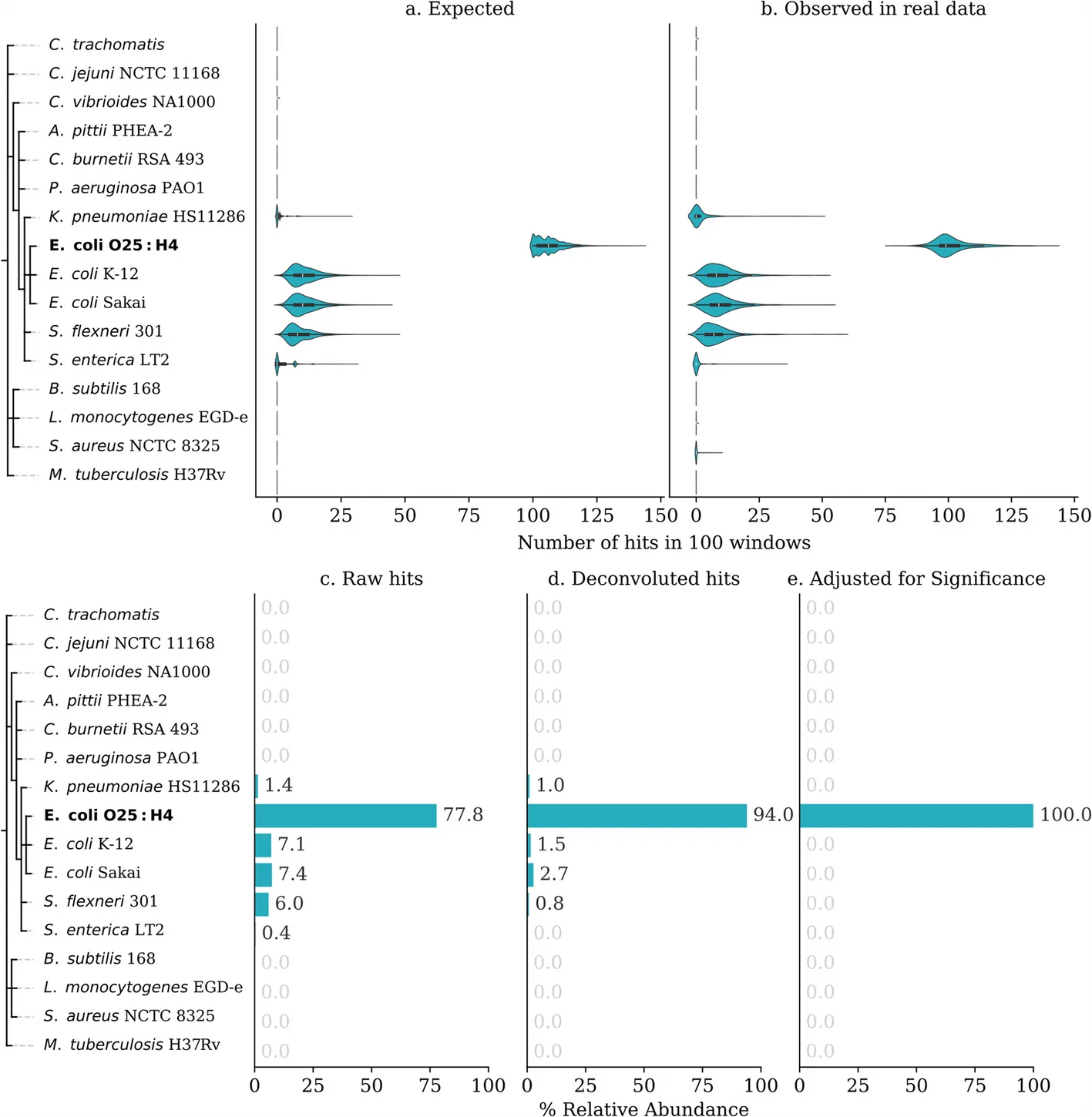
Specificity of *Voyager*’s mapping algorithm in *permutation space* and against real data. **a** Expected specificity of the *Voyager* mapping algorithm measured through 100,000 random permutations of 100 randomly chosen *vectors of ranks*, *window size* 14, motif “AST”. **b** Observed specificity in real sequencing data using random permutations in the same manner. **c** Raw hits were obtained after mapping a real sequencing sample from an *E. coli* O25:H4 isolate using the *Voyager* algorithm. **d** Hits obtained from the same sample after deconvolution was applied. **e** Hits were reported after adjusting for significance (*p*-value < 0.05). The NCBI RefSeq bacterial selected reference genomes have been used. *E. coli* O25:H4 has been added based on the ground truth information available for the sample

In the case of closely related organisms or strains, we expect a fraction of hits to multiple genomes due to sequence similarity. Rather than applying a taxonomic approach that decreases taxonomic resolution, *Voyager* aims to correct for these multiple hits by applying a deconvolution algorithm, which explicitly assumes that all the genomes are present in the database. We thus first compared the specificity of *Voyager*’s algorithm between real sequence data and the theoretical optimum, i.e., reads simulated from the genomes directly and without any sequencing errors. We first simulated perfect reads in 100 000 permutations by extracting 100 randomly chosen *vectors of ranks*, *window size* 14, motif “AST”, per permutation from the *Escherichia coli* O25:H4 genome (NCBI GenBank assembly (GCA_030507855.1), and mapped these into the 15 NCBI bacterial selected reference genomes from the RefSeq collection. Figure 4a shows the distribution of the raw hits obtained by *SMDS* mapping, where *E. coli* O25:H4, as expected, receives the highest number of hits. Two other strains of *E. coli*, K12 and Sakai, and *S. flexneri*, also match the reads, albeit with far fewer hits, caused by the high average nucleotide identity between the strains (98.69%, 98.50%, and 98.55%, respectively, which we measured through whole-genome alignments using Satsuma [23]). However, with increasing distance in SMDS (*S. enterica*: 79.45%; *K. pneumoniae*: 75.92%), the hits approach close to zero percent. *Voyager* quantifies these expected overlaps for all genomes in its index and uses this information in the deconvolution stage. Additional file 2: Table S1 shows these overlaps between *E. coli* and around 21 000 RefSeq reference genomes. We then repeated the analysis for real sequencing data from a sample obtained from strain *E. coli* O25:H4 (ENA accession ERR11001111). Figure 4b shows that the experiment performed on real sequencing data results in very similar distributions, with distributions only slightly skewed towards more false positives. This can most likely be attributed to sequencing errors affecting the motif sequences and/or the ranked order of distances between them. Additional file 1 expands on this experiment with additional *E. coli* type strains.

In quantifying specificity, the raw hits obtained from the mapping algorithm (Fig. 4c) clearly follow the expected distributions. This provides a strain level specificity of 77.8%, where most false matches occur in genomes with high genomic similarity. After *Voyager*’s deconvolution (Fig. 4d), these false matches are mostly corrected for, obtaining a 94% strain-level sensitivity. The deconvolution algorithm cannot account for individual variation in error rate and bias between sequencing runs, which is likely why it cannot correct for 100% of false hits. Finally, *Voyager* estimates the statistical significance of each genome. When correcting for this significance (*p*-value reported by Voyager < 0.05), *Voyager* obtained strain-level discrimination with 100% sensitivity in this case (Fig. 4e).

### High accuracy by correcting false positives

**Fig. 5:**
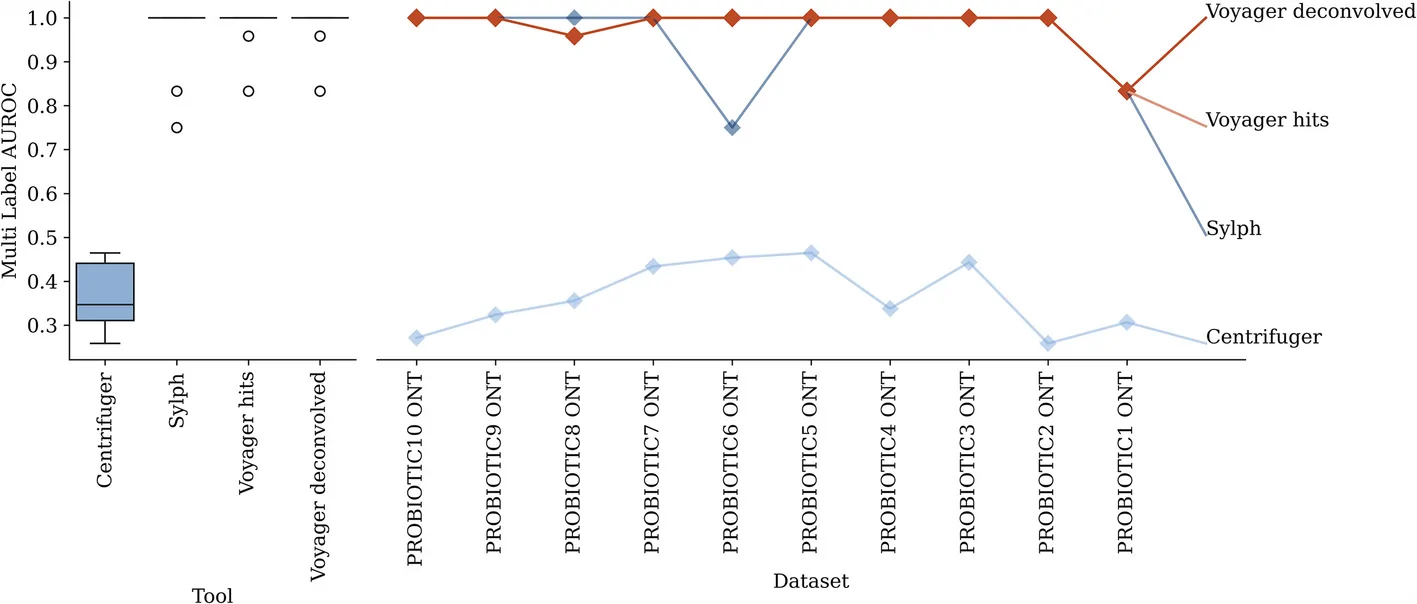
Benchmark of taxonomic detection performance. Multi-label area under the receiver operator characteristic (AUROC) classification performance using one-vs-one averaging. Tools were benchmarked against the 10 ONT probiotic datasets provided with ground truth by Van Uffelen et al. [24] using the GTDB v226 database [25]. Accuracy metrics were based on reported “hits” and “hits deconvolved” for Voyager, “taxonomic abundance” for Sylph, and “numUniqueReads” for Centrifuger at the species level. Raw data for Voyager and Sylph are provided in Additional file 4: Table S1

Voyager was benchmarked on 10 ONT benchmarking datasets with known ground truth provided by Van Uffelen et al. [24] against the Genome Taxonomy Database (GTDB) representative genomes database [25]. To evaluate the classification performance, as well as the trade-off between false positive and false negative identifications, a multi-label area under the receiver operator characteristic (AUROC) analysis was performed for each dataset (Fig. 5). In addition to AUROC, the label ranking loss, the number of incorrectly ordered species identifications weighted by the total number of species present in the dataset, and the label ranking average precision score, the average ratio of true vs. total identified species (not affected by the number of true negatives), are shown in Additional file 3. Raw results for Voyager and Sylph are provided in Additional file 4: Table S1. Results show both Voyager and Sylph provide species-level results with few false positives. False negatives for Voyager were *Lactobacillus delbrueckii* in the PROBIOTIC8 dataset, and Sylph missed *Lactiplantibacillus pentosus* in the PROBIOTIC6 dataset, highlighting differences in approach between these tools, as well as that SMDS is not a direct representation of genomic relatedness. Neither Voyager, Sylph, nor Centrifuger identified *Bifidobacterium breve* in the PROBIOTIC 1 dataset. Overall agreement between Voyager and Sylph was high across all tested datasets Additional file 4: Table S1. In addition to these results, *Voyager* was tested according to the Marić et al. [26] benchmark (see Additional file 5). Those results further highlight that Voyager achieves low false positive rates.

### Efficient mapping algorithm translates to high real-world performance

**Fig. 6:**
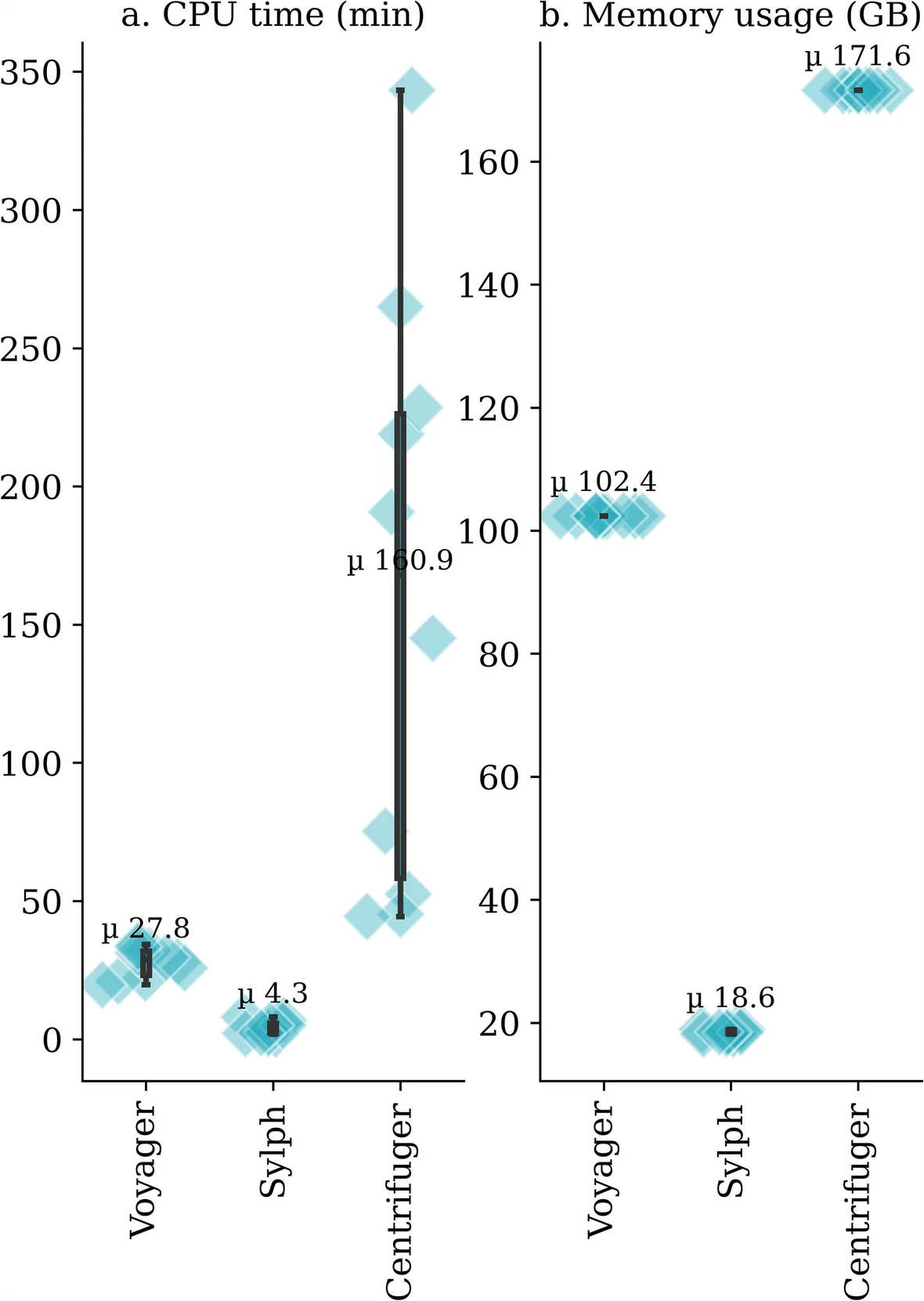
CPU time and memory usage of *Voyager,* Sylph*,* and Centrifuger. Used CPU time and peak Random Access Memory (RAM) X increase of tools for 10 long-read datasets using the GTDB database. **a** CPU time used, and **b** Peak RAM usage for each dataset. Boxplots are overlaid to visualize the quartiles and annotated with mean values

In addition to accuracy metrics, CPU time and peak random access memory (RAM) usage were measured for the above-mentioned benchmarks (Fig. 6). *Voyager* used, on average, 28 CPU minutes per dataset, while Sylph used 4, and Centrifuger used 161 CPU minutes on average, respectively. Notably, Centrifuger’s CPU time varied greatly (σ 105 min). The benchmark database index for Voyager used around 102 GB of RAM. The corresponding indices for Sylph and Centrifuger used around 19 GB and 172 GB, respectively. RAM usage varied minimally across datasets for all tested tools (σ ≤ 0.3 GB).

### Reporting and visualization

**Fig. 7:**
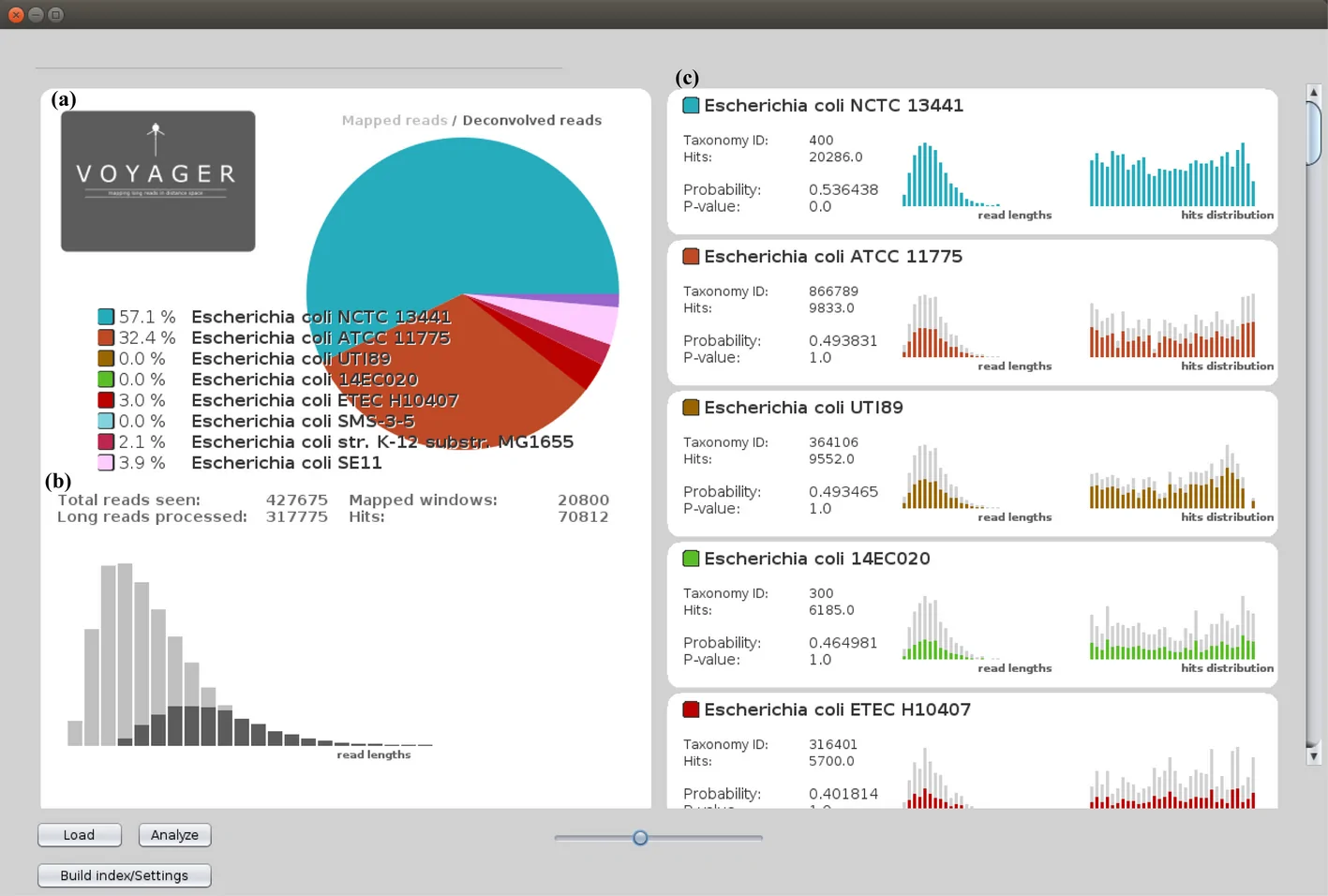
Screenshot of *Voyager Monitor*. The current taxonomic assessment is shown and updated while *Voyager* processes the data. Shown are: **a** a pie chart of the taxonomic distribution based on either the hits or the deconvoluted hits; **b** the distribution of read length, overlaid by the distribution of mapped reads (not to scale for better visual readability); and **c** a list of species detected, with the NCBI taxonomy ID, and distributions of read lengths and how they cover the respective genomes

*Voyager* allows sample profiling while keeping up with the rate of sequencing during the sequencing run. *Voyager* itself is implemented as a command line tool that can easily be integrated into other bioinformatics pipelines. The output is transferred through an API while the analysis is ongoing in a machine-readable format. To facilitate the interpretation of the results during analysis, a graphical user interface (GUI), the Voyager Monitor, is provided*,* written in Java. The GUI receives ongoing updates from *Voyager* in JSON format via a local connection and displays the current results (Fig. 7). The overview panel (left) reports the fraction of hits by genome either before or after deconvolution and the distribution of read lengths in the sample and lengths of mapped reads. The genome panel (right) shows details for each genome, the read length distribution, both absolute (light blue) and scaled on a per-genome basis (gray), as well as the spatial distribution of hits throughout the genome. Other GUI applications for visualizing sample profiling results include Pavian [27] (implemented as an R package), which includes a diagram summary of the taxonomy results, a tabular overview for multiple samples with filtering and sorting and an alignment viewer, and Recentrifuge [28], which can remove contamination when supplied with control samples, estimate confidence levels based on taxonomy information and combine multiple samples. These other methods, however, differ from *Voyager’s* approach in a number of ways. They are GUIs that enable interactive analysis of results from one or more existing tools, including taxonomy browsing, multi-sample analysis, and processing of results based on generic scoring systems. On the other hand, the Voyager Monitor receives ongoing updates, which, combined with the continuous data output enabled by ONT sequencing technology, could enable monitoring and analysis in parallel with ongoing sequencing (for a comparison of GUI features, see Additional file 2: Table S2). Furthermore, the *Monitor* can build *Voyager* indices and run analyses without interacting through a command line, potentially lowering the entry barrier for users with less bioinformatics experience [29].

## Discussion

The ultimate goal of using sequencing technology in healthcare settings is to provide clinically relevant information at minimal turnaround time, high accuracy in clinical performance, and low cost. While advances in biotechnology have significantly sped up data acquisition, a new generation of analysis tools will enable data processing in real-time while sequencing is ongoing and the sequencing machine generates data. This combination and integration allow rapid and precise diagnostics of bacterial infections, monitoring and managing disease outbreaks, and analysis of environmental samples for, e.g., surveying freshwater quality. The specific goal is to identify the presence of known organisms so that appropriate action can be taken. We would like to stress that *Voyager’s* focus lies on detecting the signatures of known genomes, and that *Voyager* is not intended to characterize novel organisms. We also point out that the selection of genomes that gets compiled into *Voyager’s* index is critical for detecting accurate signals.

*Voyager*’s algorithm further advances the field by providing a solution to the long-standing problem of processing long-read sequences, which is to rapidly and efficiently map long reads with a pattern of insertion- and deletion-errors to a large set of reference genomes. Using *Sorted Motif Distance Space* allows for a high level of compression of sequencing data while maintaining specificity. This enables *Voyager* to demonstrate high speed while maintaining relatively low memory usage. Moreover, we have shown that using *SMDS* provides very high specificity and can achieve strain-level discrimination, which is vital for, e.g., distinguishing antibiotic resistant strains or epidemiological monitoring. *Voyager*’s genome deconvolution algorithm largely corrects for false signals caused by sequence similarity and other artifacts, regardless of the underlying cause, since the expected overlaps are trained from the genomic data. We have demonstrated this method’s potential on relevant real-world datasets and shown it can obtain higher species-level classification accuracy than current tools.

One feature of *Voyager* is that the number of matches it finds drops exponentially with edits in the sequence (see Additional file 6). On one hand, this allows *Voyager* to discern between closely related species since even a small number of differences eliminates false positives. On the other hand, it also follows that sequencing errors greatly impact *Voyager’s* sensitivity, meaning not every read necessarily results in a positive identification. This needs to be compensated for by considering a larger amount of data. Furthermore, inclusion of the standardized CAMI2 metagenomic benchmark [30] was considered in addition to the presented benchmarks. However, CAMI2 datasets are simulated based on legacy ONT chemistries, and error rates of the included long-read datasets were as high as 20%, exceeding current ONT error rates by roughly 10x. Furthermore, this level of sequence divergence significantly exceeds identity requirements for genus and species level detection (97% and 99%, respectively) [31]. Since *Voyager’s* sensitivity is exponentially related to error rates, *Voyager* is intended to be used with ONT R9 (discontinued since 2024 [32]) or more recent chemistries. Error rates, in the case of ONT sequencing, have been steadily declining over the last few years, and the read quality is expected to increase even further [33]. In that regard, *Voyager*’s algorithm is “future-proof”, as with improved error rates, positive identification may become possible on a per-read basis. Lower error rates may also enable parameter changes like *window size* and motif length that could further improve *Voyager*’s efficiency.

*Voyager* is also unique because it comes with a free and easy-to-use graphical and interactive tool that allows for monitoring results and building a database of reference genomes. This intuitive interface makes *Voyager* accessible to non-experts in bioinformatics while retaining the ability to be deployed in classic bioinformatics pipelines. In addition, developers can use the API provided by *Voyager* to pass output in real-time to any application. Its goal is to provide simple yet comprehensive information about the most likely composition of the sample in true real-time. To this end, *Voyager* reports probability values, which indicate the likelihood of the sample containing that genome. In addition to this probability, *p-values* are also provided that demonstrate confidence in this probability value. With a growing need for rapid diagnostics applications, this may enable metagenomic whole genome sequencing to be more accessible in, for example, clinical settings where bioinformatics expertise has often been a barrier to adoption [29].

*Voyager* implements two main novel concepts: mapping reads in *SMDS* and using deconvolution to estimate the genomic composition of a sample. While *Voyager’s* algorithms currently provide a very fine-grained taxonomic resolution, they also have limitations. *Voyager* is currently limited to detecting organisms that are in the genomic database. If a genome in the sample is not captured in the database, *Voyager* might assign hits from these reads to closely related organisms, albeit at lower rates (exponentially decreasing with sequence divergence), resulting in a confident identification. This limitation can be remedied in a future version by estimating the expected hit counts from quality information and using this information to detect taxonomic novelty. *Voyager* was also not designed to work on small genomes or with short reads. This limitation may be remedied by combining deconvolution with other mapping algorithms, or alternatively, by considering more than one motif per sequence at the cost of increasing index size. This is also a potential way to further enhance *SMDS* discrimination. The efficiency of *Voyager’s* algorithm could make it a prime candidate for querying massive (future) databases. In this regard, pan-genome support could further improve its efficiency and avoid bias due to database imbalance by decreasing genomic redundancy. Furthermore, we expect that long-read error rates will one day be low enough so that the *SMDS* method can also be applied to, e.g., Whole Genome Assembly (WGA), both for single organisms and, in particular, for mixed-species metagenomic samples. In the meantime, profiling sample taxonomy against databases of known organisms is a prime application for *Voyager*’s algorithm because, as we have shown, *Voyager* delivers accurate results.

## Conclusions

*Voyager’s* algorithm can help address the demand for rapid, efficient, specific, and easy-to-use algorithms resulting from ever-increasing database size and complexity.

## Methods

### Read mapping

We define a soft-mer motif as an IUPAC degenerate nucleotide code of short length and its reverse complement. For example, when supplying *Voyager* with the soft-mer “AMC”, *Voyager* will look for exact matches of “AAC”, “ACC”, and their reverse complements “GTT”, “GGT”, in a given sequence and record their absolute starting positions. Once the absolute positions of exact motif matches are known in a sequence, *Voyager* constructs a *local distance vector* of that sequence. Let

$${x}_{S}\mathrm{=}\left\{\left({x}_{1},{x}_{2},\dots ,{x}_{n}\right)\text{ | }{x}_{i}\in { \, Z}_{+}^{n} \, for \, each \, i\right\}$$

be the absolute starting positions of exact matches of a soft-mer motif *M* in a sequence *S*. Then the *local distance vector* of *S*,

$${d}_{S}= \text{ } \left(\begin{array}{cccc}{x}_{2}\text{- }{x}_{1},& {x}_{3}\text{- }{x}_{2},& \dots ,& {x}_{n}\text{- }{x}_{n-1}\end{array}\right)$$

represents the local distances between consecutive matches of the motif *M* in *S*. A sliding window approach through ${d}_{S}$ with window size w is used to obtain n-1 *vectors of ranks*.

$$r \, for \, window \, m\mathrm{=}\left(\begin{array}{cccc}R\left({d}_{m}\right),& R\left({d}_{m\mathrm{+}1}\right)& \dots ,& R\left({d}_{m\mathrm{+}w}\right)\end{array}\right)$$

where $R\left({d}_{i}\right)$ is the rank of ${d}_{i}$ in the window. Given a *vector of ranks* r with window size w, we can describe a hash function

$$H\left(r\right)= \text{ } \sum\limits_{i\mathrm{=}0}^{w-1}\sum\limits_{j\mathrm{=}i\mathrm{+}1}^{w-1}\left[{r}_{j}> \text{ } {r}_{i}\right] \, \cdot \, \left({r}_{j}-1\right) \, \cdot \, \left(w-i-1\right)!$$

to represent the *vector of ranks* in a compressed form as a single positive integer. Here, $\left[{r}_{j}> \text{ } {r}_{i}\right]$ represents an indicator function which evaluates to 1 if the condition is true and 0 otherwise. To account for reverse complementarity, a window $\left[{d}_{m},{d}_{m\mathrm{+}w}\right]$ gets assigned a *sort space hash*

$$S\left({d}_{m},{d}_{m\mathrm{+}w}\right)= \text{ } min(H\left(r\right), \, H\left({r}^{R}\right))$$

where ${r}^{R}$ is the *vector of ranks* as if the window was reversed

$${r}^{R}\mathrm{=}\left(\begin{array}{cccc}R\left({d}_{m\mathrm{+}w}\right),& R\left({d}_{m\mathrm{+}(w-1)}\right)& \dots ,& R\left({d}_{m}\right)\end{array}\right)$$

and r is the *vector of ranks* of the window. Then, in *permutation space,* these *sort space hashes* are mapped to each genome, which allows rapid lookup using a hash map data structure.

### Deconvolution

Because of sequence complementarity between related genome sequences, multiple genomes are expected to share *sort space hashes*. *Voyager* quantifies these overlaps in a square matrix. Given k indexed genomes $$\left[\begin{array}{cc}{g}_{1},& {g}_{k}\end{array}\right]$$, the *overlap matrix*

$$A= \text{ } \left[\begin{array}{c}{p}_{1}\\ {p}_{2}\\ \vdots \\ {p}_{k}\end{array}\right]$$

contains *k* row vectors *p*, where

$${p}_{i}= \text{ } \left[{p}_{1}, \, {p}_{2},\dots , \, {p}_{k}\right]$$

gives the proportion of expected hits in ${g}_{1}$ through ${g}_{k}$, given genome ${g}_{i}$. Firstly, this information is used to deconvolve the raw obtained hits for sequence complementarity. The overlap between genomes observed from a sequencing sample is represented as the *vector of observed hits* b. This *vector of observed hits* can be mathematically described as a discrete convolution between the *overlap matrix *A and the underlying sample composition (*vector of deconvoluted hits)* x, $Ax\mathrm{=}b$. If sequencing errors were completely absent, *x* could be inferred from b and A using matrix inversion $x\mathrm{=}b{A}^{-1}$. However, non-linearities introduced by noise, such as sequencing errors, can cause matrix inversion to produce negative results since b may exist outside of the column space of A. To account for these possible non-linearities, x is constrained to contain strictly positive values. In this case, the *vector of deconvoluted hits* ${x \, }^{*}$ can be approximated by minimizing the Euclidean norm of ${Ax \, }^{*} \, b$ using the Householder’s transformations method for solving the NNLS problem as described by Lawson & Hanson. The NNLS algorithm takes the constraint for ${x \, }^{*}$ into account by finding $arg \, {min}_{{x}^{*}} \, \Vert {Ax \, }^{*}-b\Vert$ subject to ${x}^{*}\ge 0$(meaning all components of ${x}^{*}$ are strictly positive). If $x\mathrm{=}b{A}^{-1}$ does not produce any negative components, then NNLS and matrix inversion give the same results. If not, however, then NNLS computes the best fit given the constraint. To optimize efficiency of the deconvolution step, the overlap matrix is subset to exclude genomes with no hits before running deconvolution. This ensures the speed is not limited by the database size, but rather by the number of genomes in the sample.

### Statistical significance estimation

*Voyager* also uses this *overlap matrix* to build a likelihood model for each of the genomes in the database, as well as a null hypothesis model. The null hypothesis model assumes that the proportions of observed hits are random and thus are distributed according to the respective proportion of each genome in *permutation space*. The alternative model assumes that the observed hits are distributed according to ${p}_{i}$. *Voyager* computes the log likelihood of such a model θ is given a *vector of hits* X as

$$log\left(P\left(\theta \text{ | }X\right)\right)= \sum\limits_{i\text{ = }0}^{k}{x}_{i}\cdot \, log({p}_{i})$$

Then, the probability of the genome can be derived from the likelihood of the null hypothesis model and the genome model according to

$$P\left({\theta}_{genome}\right)\mathrm{=}\frac{P\left({\theta}_{genome}\mathrm{|}X\right)}{P\left({\theta}_{null \, hypothesis}\mathrm{|}X\right)+ \text{ } P\left({\theta}_{genome}\mathrm{|}X\right)}$$

The corresponding *p*-value is computed using the chi^2^ cumulative distribution function $chisq\mathrm{\_}cdf$, and the likelihood ratio *LR* as follows: *p*-value $\text{ }1-chisq_{cdf}\left(LR;DDOF\right)$, where

$$LR\mathrm{=}\frac{2\left(log\left(P\left({\theta}_{genome}\mathrm{|}X\right)\right)-log\left(P\left({\theta}_{null \, hypothesis}\mathrm{|}X\right)\right)\right)}{20}$$

and

$$DDOF\mathrm{=}k-1$$

The $chisq_{cdf}$ is computed using the lower incomplete gamma series expansion method as

$${chisq}_{cdf\left(a, \, x\right)}= \frac{1}{a}\sum\limits_{i\mathrm{=}0}^{\infty }\frac{{x}_{i}}{\left(a\mathrm{+}i\right)!}$$

until convergence or the iteration threshold is reached. *P*-values are then adjusted for multiple testing using the Holm-Bonferroni method [34]. Briefly, the *p*-values are ranked from smallest to largest, and the adjusted *p*-value is the maximum of the previous *p*-value and the *p*-value multiplied by, $m\mathrm{+}1-k$ where *m* is the number of tested hypotheses and *k* is the rank of the *p*-value.

### Benchmarking

Benchmarking datasets with known ground truth species composition were taken from Van Uffelen et al. [24] and deposited in the Sequence Read Archive under BioProject accession number PRJNA1334416. Briefly, ten mock communities were derived from commercial probiotic products and sequenced using the SQK-LSK114 ligation kit on an individual R10.4.1 MinION flow cell per individual mock community. Each tool was benchmarked using the representative genomes of the GTDB release 10-rs226 database [25], and all indices were built using the respective tools’ default parameters. The following software versions were used: Voyager v0.1.6, Sylph v0.9.0, Centrifuger v1.1.0-r299. CPU time and memory usage were measured using the Linux time command. All benchmarks were performed on a server with dual 64-core Intel Xeon E5-2697 v4 2.30 GHz CPUs and 1 TiB of DDR4 memory. Accuracy metrics were based on reported “hits” and “hits deconvolved” for Voyager, “taxonomic abundance” for Sylph, and “numUniqueReads” for Centrifuger at the species level, which were converted to percentages. Accuracy metrics, multi-label area under the receiver operator characteristic with one-vs-one averaging, label ranking loss, and label ranking average precision, were computed using scikit-learn v1.3.2 [35].

## Supplementary Information

Additional file 1. Details about the *E. coli* genomes downloaded from NCBI.

Additional file 2: Table S1. Number and fraction of overlapping windows between *E.coli* and Refseq genomes. Table S2. Information about additional benchmarks. Table S3. Raw benchmarking results. Table S4. CPU and memory usage.

Additional file 3. Benchmark of taxonomic binning.

Additional file 4: Table S1. Suppressed Genomes from the NCBI database and replacements.

Additional file 5. Details about the Benchmarking datasets.

Additional file 6. Parametrization in Sorted Motif Distance Space.

## Data Availability

Voyager is open-source software released under the GNU Lesser General Public License v3.0 (https://www.gnu.org/licenses/lgpl-3.0.en.html). Voyager is available from Bitbucket https://bitbucket.org/sverre-phd-work/voyager/src/master/ [36]; Bioconda https://anaconda.org/channels/bioconda/packages/voyager/overview [37] and Zenodo https://doi.org/10.5281/zenodo.21508803 [38]. Voyager is also available via bioconda (bioconda::voyager).
