# Supplementary material for: Voyager: rapid taxonomic characterization of long-read mixed-species sequencing samples in sorted motif distance space
Source: Genome Biol. 2026 Aug 5;27:340. doi: 10.1186/s13059-026-04235-3 (PMC13726117; doi:10.1186/s13059-026-04235-3)
Supplement: Supplementary file 1 [file 13059_2026_4235_MOESM1_ESM.docx]

**Details about the complete and chromosome level assemblies downloaded for *Escherichia coli* type strains (selecting ‘From type material’) from NCBI.**

| **Type strain** | **Assembly accession** |
| --- | --- |
| 14EC020 | ASM285371v1 |
| UTI89 | ASM1326v1 |
| ATCC 11775 | ASM369716v2 |
| ETEC H10407 | ASM21047v1 |
| SE11 | ASM1038v1 |
| SMS-3-5 | ASM1964v1 |

**The *E. coli* reference strains and the ground truth strain reference assemblies.**

| **Strain** | **Assembly accession** |
| --- | --- |
| K-12 | ASM584v2 |
| Sakai | ASM886v2 |
| O25:H4 | 32512_F01 |

The genomes were indexed using default parameters: motif: CWG, window size: 14, minimum length: 512.

Taxonomic profiling was done for sequencing reads from an *E. coli* O25:H4 strain (ENA accession ERR12778389) using this index.

**Information about the specificity of the profiling**

| **Genome** | **SMDS**  **hits** | **Deconvolved**  **hits** | **Relative**  **SMDS**  **hits** | **Relative**  **deconvolved**  **hits** |
| --- | --- | --- | --- | --- |
| ***Escherichia coli* O25:H4** | 20286 | 37843.00125 | 1 | 1 |
| *Escherichia coli* ATCC 11775 | 9833 | 21473.51396 | 0.484718 | 0.567436 |
| *Escherichia coli* SE11 | 4155 | 2610.83347 | 0.204821 | 0.068991 |
| *Escherichia coli* ETEC H10407 | 5700 | 2017.874429 | 0.280981 | 0.053322 |
| *Escherichia coli* str. K-12 substr. MG1655 | 4802 | 1402.244055 | 0.236714 | 0.037054 |
| *Escherichia coli* Sakai | 4060 | 914.094775 | 0.200138 | 0.024154 |
| *Escherichia coli* UTI89 | 9552 | 0 | 0.470866 | 0 |
| *Escherichia coli* 14EC020 | 6185 | 0 | 0.304890 | 0 |
| *Escherichia coli* SMS-3-5 | 5211 | 0 | 0.256876 | 0 |
| *Escherichia coli* SMS-3-5 plasmid | 267 | 0 | 0.013161 | 0 |
| *Escherichia coli* ETEC H10407 plasmid | 256 | 0 | 0.012619 | 0 |
| *Escherichia coli* 14EC020 plasmid | 246 | 0 | 0.012126 | 0 |
| *Escherichia coli* ATCC 11775 plasmid | 104 | 0 | 0.005126 | 0 |
| *Escherichia coli* UTI89 plasmid | 100 | 0 | 0.004929 | 0 |
