# Supplementary material for: Voyager: rapid taxonomic characterization of long-read mixed-species sequencing samples in sorted motif distance space
Source: Genome Biol. 2026 Aug 5;27:340. doi: 10.1186/s13059-026-04235-3 (PMC13726117; doi:10.1186/s13059-026-04235-3)
Supplement: Supplementary file 2 [file 13059_2026_4235_MOESM3_ESM.docx]

**Benchmark of taxonomic binning performance.** Tools were benchmarked against the 10 ONT probiotic datasets provided with ground truth by Van Uffelen, et al. 2026 (23) using the GTDB v226 database (24). Accuracy metrics were based on reported “hits” and “hits deconvolved” for Voyager, “taxonomic abundance” for Sylph, and “numUniqueReads” for Centrifuger at species level. 1 - label ranking loss (a) represents the number of incorrectly ordered species identifications weighted by the total number of species present in the dataset. Label ranking average precision (b) shows the average ratio of true vs. total identified species.
