# Supplementary material for: Voyager: rapid taxonomic characterization of long-read mixed-species sequencing samples in sorted motif distance space
Source: Genome Biol. 2026 Aug 5;27:340. doi: 10.1186/s13059-026-04235-3 (PMC13726117; doi:10.1186/s13059-026-04235-3)
Supplement: Supplementary file 3 [file 13059_2026_4235_MOESM5_ESM.docx]

Benchmarking datasets with known ground truth species composition were taken from Maric et al. (22). Briefly, these datasets include mock communities, as well as in-silico datasets obtained by mixing real reads from isolated, sequenced species. In addition, clinically relevant datasets with known round truth, and a ONT R10.3 mock community sample were retrieved from ENA (see Table s1 for more details about the datasets). These datasets ensure taxonomic divergence from the database reference genomes and more realistic error rates and patterns compared to simulated datasets such as the CAMI II long read dataset. The benchmarking database contained NCBI-NT bacteria and archaea with ‘Complete Genome’ or ‘Chromosome’ level assemblies as described (22). Minor deviations due to genomes being ‘suppressed’ by NCBI are documented in Table s2. True positives (TP), false positives (FP), etc. were identified according to Maric et al.’s organism detection methods using a 1 read threshold. Any genomes identified by any tool, but not present in the sample were considered true negatives (TN). Precision, Recall, Accuracy and F1 score were calculated as described. Where possible, benchmarking results from Maric et al. were used directly. The following software versions were used to obtain additional results: bracken v2.9, centrifuge v1.0.4, centrifuge v1.0.5-r170, mashmap v3.1.3, kraken v2.1.3. CPU time and memory usage were measured using the Linux time command. All benchmarks were performed on a server with dual 64 core Intel Xeon E5-2697 v4 2.30 GHz CPUs and 1 TiB of DDR4 memory.

Table s1: Overview of additional benchmarking datasets

| **name** | **dataType** | **accessions** | **Publication DOI** | **groundTruth** |  |
| --- | --- | --- | --- | --- | --- |
|  |  |  |  | **speciesName** | **strainName** |
| Blood Flongle | ONT Flongle R9.4.1 | ERR7565106 | 10.3389/fmicb.2022.822402 | Escherichia coli | Escherichia coli 125 |
|  |  | ERR7565107 |  |  |  |
|  |  | ERR7565108 |  |  |  |
|  |  | ERR7565109 |  |  |  |
|  |  | ERR7565110 |  |  | Escherichia coli A2-39 |
|  |  | ERR7565111 |  |  |  |
|  |  | ERR7565112 |  |  |  |
|  |  | ERR7565113 |  | Klebsiella pneumoniae | Klebsiella pneumoniae 225T |
|  |  | ERR7565114 |  |  |  |
|  |  | ERR7565115 |  |  |  |
|  |  | ERR7565116 |  |  |  |
|  |  | ERR7565117 |  |  | Klebsiella pneumoniae A2-37 |
|  |  | ERR7565118 |  |  |  |
|  |  | ERR7565119 |  |  |  |
|  |  | ERR7565120 |  |  |  |
| Blood MinION | ONT MinION R9.4.1 FLO-MIN106 | ERR11001110 | 10.1038/s41598-020-64616-x | Escherichia coli | Escherichia coli 125 |
|  |  | ERR11001111 |  |  | Escherichia coli A2-39 |
|  |  | ERR11001112 |  |  | Escherichia coli NCTC 13441 |
|  |  | ERR11001113 |  | Klebsiella pneumoniae | Klebsiella pneumoniae A2-23 |
|  |  | ERR11001114 |  |  | Klebsiella pneumoniae A2-37 |
|  |  | ERR11001115 |  | Staphylococcus aureus | Staphylococcus aureus CCUG 35600 |
|  |  | ERR11001116 |  |  |  |
| Milk | ONT MinION R9.4.1 FLO-MIN106 | ERR9847751 | 10.3389/fmicb.2022.1104701 | Staphylococcus aureus |  |
|  |  | ERR9847752 |  |  |  |
|  |  | ERR9847753 |  |  |  |
|  |  | ERR9847754 |  |  |  |
|  |  | ERR9847755 |  | Bos taurus |  |
|  |  | ERR9847756 |  |  |  |
|  |  | ERR9847757 |  |  |  |
| Urine | ONT MinION R9.4.1 FLO-MIN106 | ERR12778389 | 10.1101/2024.03.04.582689 | Escherichia coli | Escherichia coli INN1 |
|  |  | ERR12778390 |  |  | Escherichia coli INN2 |
|  |  | ERR12778391 |  |  | Escherichia coli INN3 |
|  |  | ERR12778392 |  |  | Escherichia coli NCTC 13441 |
|  |  | ERR12778393 |  | Enterococcus faecalis | Enterococcus faecalis CCUG 9997 |
|  |  | ERR12778394 |  | Klebsiella pneumoniae | Klebsiella pneumoniae CCUG 225T |
|  |  | ERR12778395 |  | Pseudomonas aeruginosa | Pseudomonas aeruginosa CCUG 19619 |
|  |  | ERR12778396 |  | Proteus mirabilis | Proteus mirabilis CCUG 26767T |
|  |  | ERR12778397 |  | Staphylococcus aureus | Staphylococcus aureus NCTC 8325 |
| Zymo R10.3 | ONT GridION R10.3 | https://nanopore.s3.climb.ac.uk/mock/Zymo-GridION-EVEN-3Peaks-R103-merged.fq.gz | 10.1128/msystems.00190-20 | Listeria monocytogenes |  |
|  |  |  |  | Pseudomonas aeruginosa |  |
|  |  |  |  | Bacillus subtilis |  |
|  |  |  |  | Escherichia coli |  |
|  |  |  |  | Salmonella enterica |  |
|  |  |  |  | Lactobacillus fermentum |  |
|  |  |  |  | Enterococcus faecalis |  |
|  |  |  |  | Staphylococcus aureus |  |
|  |  |  |  | Saccharomyces cerevisiae |  |
|  |  |  |  | Cryptococcus neoformans |  |

Table s2: Database substitutions

| **suppressed** | **new** |
| --- | --- |
| GCF_000017125.1 | GCF_000013425.1 |
| GCF_000014905.1 | NA |
| GCF_000016865.1 | GCF_000412675.1 |
| GCF_000022045.1 | NA |
| GCF_000025585.1 | GCF_000830905.1 |
| GCF_000013105.1 | GCF_001267435.1 |
| GCF_000177255.2 | GCF_016584445.1 |
| GCF_000016365.1 | GCF_000184435.1 |
| GCF_005484925.1 | NA |
| GCF_000019745.1 | GCF_900096765.1 |
| GCF_000016565.1 | GCF_000733715.2 |
| GCF_000012585.1 | NA |
| GCF_000016705.1 | GCF_000830905.1 |
| GCF_000023345.1 | GCF_033807855.1 |
| GCF_000092605.1 | GCF_002028405.1 |
| GCF_000019145.1 | NA |
| GCF_000016585.1 | GCF_025402875.1 |
| GCF_000018265.1 | GCF_000384275.1 |
| GCF_000486165.2 | GCF_000006945.2 |
| GCF_000176935.3 | NA |
| GCF_000183525.2 | GCF_000621585.1 |
| GCF_000495635.1 | GCF_000495635.2 |
| GCF_000195835.2 | GCF_000195955.2 |
| GCF_000175115.2 | NA |
| GCF_000148685.1 | NA |
| GCF_000024045.1 | GCF_000021805.1 |
| GCF_000023645.1 | NA |
| GCF_000012605.1 | GCF_004786015.1 |
| GCF_000020205.1 | GCF_902374465.1 |
| GCF_000022185.1 | GCF_000018865.1 |
| GCF_000014265.1 | NA |
| GCF_000148425.1 | NA |
| GCF_000175095.2 | NA |
| GCF_000015405.1 | NA |
| GCF_000022125.1 | GCF_017815575.1 |
| GCF_000169215.2 | GCF_025402875.1 |
| GCF_000014825.1 | GCF_016584445.1 |
| GCF_000014665.1 | NA |
| GCF_000016885.1 | NA |
| GCF_000203875.1 | GCF_019602855.1 |
| GCF_000014245.1 | NA |
| GCF_902143595.1 | NA |
| GCF_000024165.1 | GCF_012939955.1 |
| GCF_000019445.1 | GCF_000412675.1 |
| GCF_000024705.1 | NA |
| GCF_000013745.1 | GCF_016584445.1 |
| GCF_000011785.1 | GCF_000019405.1 |
| GCF_000019285.1 | GCF_018135955.1 |
| GCF_000147855.2 | GCF_000005845.2 |
| GCF_000020085.1 | GCF_000834295.1 |
| GCF_000012425.1 | NA |
| GCF_000702925.2 | GCF_016865255.1 |
| GCF_000014085.1 | GCF_000019505.1 |
| GCF_000023385.1 | NA |
| GCF_000012405.1 | GCF_015034585.1 |
| GCF_000023425.1 | GCF_902374465.1 |
| GCF_000016085.1 | GCF_003968655.1 |
| GCF_000017285.1 | NA |
| GCF_000176115.2 | GCF_016804005.1 |
| GCF_007291315.1 | GCF_902387845.1 |
| GCF_000019465.1 | GCF_000834295.1 |
| GCF_000018085.1 | GCF_949794035.1 |
| GCF_000021385.1 | GCF_000015485.1 |
| GCF_009663835.1 | GCF_000005845.2 |
| GCF_000192845.1 | NA |
| GCF_000020545.1 | GCF_000015125.1 |

**Results** *Voyager* was benchmarked against the state-of-the-art long-read metagenomic classifiers, according to Marić et al. (2024) (22). Briefly, the benchmark includes 7 in-silico community datasets constructed by mixing real reads from isolated sequenced species and 3 mock community datasets. The datasets contain ONT or PacBio data from bacterial, archaeal, and eukaryotic species, some with even distributions and some with decreasing ratios. The database was constructed using NCBI-NT sequences from RefSeq as described (22). Genomes that were no longer available (suppressed) from NCBI were replaced by corresponding updated reference genomes where possible (see Table s2). The V*oyager* index was made with default settings (motif CWG, window size 14, minimum length 512). All metrics were obtained at the species level with a read threshold of 1 as described (22). *Voyager* was run with default parameters.

Figure s1: Species level classification accuracy and F1 score according to Marić et al. Metrics were calculated on a per-species basis, according to Maric et al.’s organism detection methods using a 1 read threshold. (a) Accuracy score. (b) F1 score. Data for tools other than *Voyager* were taken from Marić et al. (22). The boxplot (left) shows the quartiles across all datasets. Outliers are not shown.

Raw hits obtained from the *Voyager SMDS* mapping algorithm resulted in an average classification accuracy of 82.6% across the 10 datasets (Figure s1a). The relatively low classification accuracy can be mainly attributed to a high false positive rate (mean precision 3.2%), comparable to k-mer based methods (mean precision for Centrifuge, Centrifuger, and Kraken2 respectively 3.7%, 5.3%, and 2.4%). The *Voyager* deconvolution algorithm largely corrects these false positives, significantly improving precision (p-value 6.2 10^-6^) to a mean precision of 49.2 %. This is reflected in the accuracy score for *Voyager* with deconvolution (mean: 99.2%). Furthermore, the accuracy score is high (> 99%) across datasets and sequencing platforms except for dataset PB ATCC (accuracy 95.5 %). The F1 scores (Figure s1b) reflect the same findings. While these results highlight *Voyager’s* ability to accurately identify species from mixed species samples, *Voyager* does not attempt abundance estimation in its current implementation. Another caveat is that (due to the large database used) false negatives are not reflected as strongly as false positives in the accuracy scores. Deconvolution can potentially increase false positive rates by erroneously removing genomes with a low number of hits due to very low abundance and other biases. Another limitation of this benchmark is that mixing reads from different sequencing runs.

In addition to the 10 datasets from Marić et al., *Voyager* was benchmarked on 4 ONT datasets of clinically relevant sample types with known ground truth (4,23–25), and a mock community sample (26) sequenced using the more recent ONT R10.3 chemistry with improved read accuracy (see Table s1 for more details). *Voyager* was compared to kmer-based tools as they are considered most well-suited for rapid detection applications (22). MashMap was included since MinHash-based methods were lacking from the benchmarking results. The database and methods used were based on benchmarks by Marić et al. (22) as described in the Methods section. Results for these datasets (Figure s2) further confirm the previous findings; raw *SMDS* results in a high false positive rate (mean precision 0.4%), comparable to kmer-based methods (Centrifuge, Centrifuger, and Kraken2 mean precision 0.3%, 0.3%, and 0.5%, respectively), which results in a mean accuracy score of 69.7%. Subsequently, deconvolution corrects for a lot of false positives, increasing mean precision to 54.6% and mean accuracy score to 99.7%. *Voyager’s* accuracy score exceeded 99% for all datasets. The Milk dataset was the most challenging dataset for every tool, where false positive rates were high across the board (as reflected in Figure s2b), likely due to the high proportion of contaminating host (*Bos taurus*) data. Raw performance and diversity metrics are enclosed in Additional file 2: Table S3.

Figure s2: Species level classification accuracy and F1 score of additional datasets according to Marić et al. Metrics were calculated on a per-species basis, according to Maric et al.’s organism detection methods using a 1 read threshold. (a) Accuracy score. (b) F1 score. The boxplot (left) shows the quartiles across all datasets. Outliers are not shown.

In addition to accuracy metrics, CPU time and peak random access memory (RAM) usage were measured for the above-mentioned benchmarks. *Voyager’s* CPU time and memory usage were compared to Centrifuge and Kraken2 as they are among the fastest current methods and considered the tools of choice in most scenarios (26). On average, *Voyager* used 5.09X less CPU time than Centrifuge, 12.72X less CPU time than Centrifuger, and a CPU time comparable to Kraken2 (on average 1.12X less) (Figure s3a). Differences in CPU time with Centrifuge and Centrifuger varied greatly (σ 1.54 and σ 5.09), where *Voyager* was at most 7.59X faster and at the lowest 2.53X faster than Centrifuge and *Voyager* was at most 22.9X faster and at the lowest 1.6X faster than Centrifuger. Differences in CPU time with Kraken2 fell within 0.77X and 2.49X (σ 0.30). For the 15 datasets, *Voyager* and Kraken2 used, on average, around 5 CPU minutes per dataset, while the average CPU time for Centrifuge was close to 30 minutes. Centrifuger used on average 75 minutes. The benchmark database index for Voyager used around 154 GB of RAM. The corresponding indices for Centrifuge, Centrifuger, and Kraken2 used around 385 GB, 239 GB, and 343 GB, respectively. On average, Voyager used 2.24X (σ 0.024), 1.55X (σ 0.002), and 2.50X (σ 0.006) less RAM than Centrifuge, Centrifuger, and Kraken2, respectively, analyzing the 15 datasets (Figure s3b). While *Voyager* uses single-threaded sequential indexing, it constructed the benchmarking index in under 1 hour, whereas Centrifuge, Centrifuger, and Kraken2 used around 16.5, 28.5, and 6 hours, respectively, using 20 parallel threads. Furthermore, Voyager’s sequential indexing was much faster and more memory efficient at indexing a variety of databases compared to the parallel indexing of both Centrifuge and Kraken2 using 20 parallel threads (see Additional file 2: Table S4).

Figure s3: CPU Time and Memory usage of Centrifuge, Centrifuger, and Kraken2 compared to *Voyager*. Used CPU time and peak Random Access Memory (RAM) X increase of tools compared to *Voyager* for 15 long-read datasets. (a) CPU time increases for each dataset compared to Voyager, which uses the same database for Centrifuge, Centrifuger, and Kraken2. (b) Peak RAM usage X increases for each dataset compared to *Voyager* using the same database for Centrifuge, Centrifuger, and Kraken2. Boxplots are overlayed to visualize the quartiles.
