# Supplementary material for: Voyager: rapid taxonomic characterization of long-read mixed-species sequencing samples in sorted motif distance space
Source: Genome Biol. 2026 Aug 5;27:340. doi: 10.1186/s13059-026-04235-3 (PMC13726117; doi:10.1186/s13059-026-04235-3)
Supplement: Supplementary file 4 [file 13059_2026_4235_MOESM6_ESM.docx]

**Parametrization in Sorted Motif Distance Space**

Sorted Motif Distance Space (SMDS) is fundamentally different from other representations such as k-mers or the Burrows–Wheeler transform, whereas it shares some design features with the latter. SMDS, given a nucleotide motif, uses the distances between the occurrences of the motifs, ranks them in the order in which they are found, sorts them, and uses the vector of sorted ranks as a hash. In the following, we discuss the properties of SDMS and the effect of parameter choice on the expected outcome.

**1. Sensitivity to errors**

Sorted motif vectors can be affected by sequencing errors in three ways: (a) errors that destroy motifs, where even a single missing motif will alter the vector such that it is no longer recognizable; (b) erroneous motifs created by errors; or (c) large deletions or insertions that change the sorted order.

Given a motif size m and n the number of elements in the vector, the probability of a vector remaining intact in presence of errors is

p = r^(m*n)^ * s^(n*d)^ * h^n^

where *r* is the read accuracy, *d* the average distance between motifs, and *s* the probability of a motif being created by errors, and *h* the probability of indels affecting the sorted order, where both *s* and *h* are a function of the genomic sequence, e.g. GC content etc.

Figure s1 shows the log fraction of intact vectors from simulated reads from the E. coli genome, given the motif CWG, a vector size of 14, and the measured average distance between motifs 38 nucleotides, showing the expected exponential drop in sensitivity over increasing error rates. Regression shows that this simulated drop is almost perfectly logarithmic with an R^2^ of 0.997.

**Figure s1.** Fraction of vectors detected over simulated error rates on a log scale, showing an exponential drop**.**

If only taking into account the known error rate, then, for example, we expect a value of 10.83 for 5% errors, yet the observed rate is 8.12, indicating that all three factors contribute to generating false negatives.

**2. Species specificity**

This exponential drop in sensitivity, however, also applies to genomic differences between genomes, thus providing high specificity when discerning between strains or closely related organisms.

**3. Randomness in SMDS**

The optimal representation of a search space is one in which elements are evenly distributed. In k-mer analyses for example, which rely on localized sequences, the nucleotide following any given k-mer is dependent on the sequence of the k-mer, notably last nucleotide in the k-mer. Figure s2 shows this bias for E. coli (Figure s2a) and human chromosome 1 (Figure s2b).

**Figure s2.** Frequency distribution of nucleotides following a k-mer (y axis) ending in a particular nucleotide (x axis), for (a) E. coli and (b) human chromosome 1.

The less biased a representation of a genome is, the more efficient the use of the hash space. We thus examined how “random” the distributions SDMS are: when scanning the genome for adjacent overlapping sorted vectors, we measured the frequency by which position in the vector the next motif is inserted. Specifically, we:

1) compute a sorted motif vector at one position, then

2) remove the first motif distance from the vector, and

3) move to the next motif position and add the distance to the vector,

4) sort the vector, and record in which position in the vector the newly added position is inserted

5) repeat steps 1 through 4 over the entire sequence

Using the motif CWG, vector size 14, and using a sorting algorithm that assigns elements at random in case of ties, Figure s3 shows that there is little bias in SDMS compared to the ideal flat distribution, both for *E. coli* (Figure 3sa) and human chromosome 1 (Figure s3b).

**Figure s3**. Insertion position fraction of a vector in SMDS when sliding vectors across a genome, (a) *E. coli* and (b) human chromosome 1, using motif CWG and a window size of 14.
